# Effects of Elevated CO_2_ and N Addition on Growth and N_2_ Fixation of a Legume Subshrub (*Caragana microphylla* Lam.) in Temperate Grassland in China

**DOI:** 10.1371/journal.pone.0026842

**Published:** 2011-10-26

**Authors:** Lin Zhang, Dongxiu Wu, Huiqiu Shi, Canjuan Zhang, Xiaoyun Zhan, Shuangxi Zhou

**Affiliations:** 1 State Key Laboratory of Vegetation and Environmental Change, Institute of Botany, Chinese Academy of Sciences, Xiangshan, Beijing, China; 2 Graduate School of Chinese Academy of Sciences, Yuquanlu, Beijing, China; Cairo University, Egypt

## Abstract

It is well demonstrated that the responses of plants to elevated atmospheric CO_2_ concentration are species-specific and dependent on environmental conditions. We investigated the responses of a subshrub legume species, *Caragana microphylla* Lam., to elevated CO_2_ and nitrogen (N) addition using open-top chambers in a semiarid temperate grassland in northern China for three years. Measured variables include leaf photosynthetic rate, shoot biomass, root biomass, symbiotic nitrogenase activity, and leaf N content. Symbiotic nitrogenase activity was determined by the C_2_H_2_ reduction method. Elevated CO_2_ enhanced photosynthesis and shoot biomass by 83% and 25%, respectively, and the enhancement of shoot biomass was significant only at a high N concentration. In addition, the photosynthetic capacity of *C. microphylla* did not show down-regulation under elevated CO_2_. Elevated CO_2_ had no significant effect on root biomass, symbiotic nitrogenase activity and leaf N content. Under elevated CO_2_, N addition stimulated photosynthesis and shoot biomass. By contrast, N addition strongly inhibited symbiotic nitrogenase activity and slightly increased leaf N content of *C. microphylla* under both CO_2_ levels, and had no significant effect on root biomass. The effect of elevated CO_2_ and N addition on *C. microphylla* did not show interannual variation, except for the effect of N addition on leaf N content. These results indicate that shoot growth of *C. microphylla* is more sensitive to elevated CO_2_ than is root growth. The stimulation of shoot growth of *C. microphylla* under elevated CO_2_ or N addition is not associated with changes in N_2_-fixation. Additionally, elevated CO_2_ and N addition interacted to affect shoot growth of *C. microphylla* with a stimulatory effect occurring only under combination of these two factors.

## Introduction

Increasing atmospheric carbon dioxide (CO_2_) concentration caused by combustion of fossil fuels and enhanced nitrogen (N) deposition by human activities are two factors associated with global climate change. These factors are likely to have a widespread influence on individual plant communities and their interactions with each other [1]. It is well documented that the increase in atmospheric CO_2_ concentration stimulates photosynthesis, plant biomass and plant water-use efficiency in many plant species, and that these effects are dependent on plant species as well as nutrient availability [2], [3]. It is hypothesized that the sustainability of ecosystem response to CO_2_ will be constrained by the progressive N limitation induced by the growth stimulation of increased CO_2_ [4], [5]. Therefore, the interaction between CO_2_ and soil N availability has attracted intense interest, and varying results have been reported in different studies [3], [6], [7]. For example, the results from a six-year field study of perennial grassland species showed that the positive effect of CO_2_ without N addition is reduced substantially [5]. However, plant growth in scrub-oak woodland showed a sustained increase after 11 years of atmospheric CO_2_ enrichment with enhanced inorganic N absorption from deep soil [6].

Differential sensitivities of different plant species or functional groups in response to elevated CO_2_ are often observed. Legumes show greater response to elevated CO_2_ through symbiotic N_2_ fixation to counteract the progressive N limitation than any other functional types in most cases [8]. Lee *et al* (2003) reported that the legume species *Lupinus perennis* showed a stronger response to elevated CO_2_ than non-leguminous species independent of N status, and that a 47% greater proportion of N was derived from stimulated N_2_ fixation relative to other sources of N at elevated CO_2_ [9]. The significant stimulation of N_2_ fixation by elevated CO_2_ is also reported in *Trifolium repens* in a fertilized Swiss grassland [10], *Galactia elliottii* in Florida scrub oak [11], soybean [8] and alfalfa [12]. The positive effect of elevated CO_2_ on N_2_ fixation may also contribute to the positive response of the co-occurring non-leguminous plants in response to elevated CO_2_ [13]. However, stimulation of N_2_ fixation by CO_2_ can only occur under conditions in which other nutrients (e.g. P, K, and Mg) are not limited [14], [15], [16]. Furthermore, the stimulatory effect of elevated CO_2_ on N_2_ fixation has been found to diminish with the extended period of CO_2_ enrichment in oak woodland [15]. In addition, the response of legumes and symbiotic N_2_ fixation to elevated CO_2_ is species-specific and dependent on N availability in the soil [17], [18]. Fixation of N_2_ is often suppressed by N fertilization [19], [20], but not in all cases [21]. It is predicted that plants would fix N_2_ by symbiosis under conditions where it is less costly than soil N uptake [22], and show a significant yield response to N addition when the N_2_ fixation apparatus unable to meet plant N demand [20], [23]. Furthermore, how elevated CO_2_ affects the suppression of N fixation with N addition remains unclear, but varies from no effect [16] to a positive effect [19]. In addition, elevated CO_2_ partly promotes shrub encroachment in arid or semiarid grasslands [24]. As most shrubs are C_3_ plants, they may benefit relatively more from higher levels of CO_2_ compared to many C_4_ grasses [25]. Elevated CO_2_ may slow soil water depletion by herbaceous vegetation, thus promoting the establishment of deeper-rooted shrubs, especially in semiarid and/or arid areas [26], [27], [28]. Although an increase in woody plant density was observed after CO_2_ enrichment for five years in semiarid shortgrass steppe in Colorado [24], it remains unclear whether elevated atmospheric CO_2_ plays a widespread role in encroachment of C_3_ shrub and woody plants into grasslands [26], [29].

The leguminous sub-shrub *Caragana microphylla* is a common species that dominates an important plant successional stage in the semiarid grasslands in northern China. It is reported that the distribution of *C. microphylla* shrubs in the Xilin River Basin in northern China has increased substantially in recent years [30], [31], [32]. This study was conducted to determine how the growth and symbiotic N_2_ fixation of *C. microphylla* respond to elevated CO_2_ and N addition in a semiarid temperate grassland over three growing seasons.

## Materials and Methods

### Research site

The experiment was conducted at the Inner Mongolia Grassland Ecosystem Research Station (IMGERS) (43°38′N, 116°42′E; 1100 m altitude) in the Xilin River Basin, Inner Mongolia, China. The site is located in the Eurasian steppe region, which is the largest contiguous grassland in the world. The site has a continental, moderate temperate, semiarid climate characterized by long, cool, dry winters and short, warm, moist summers. The mean annual temperature is 0.8°C, and the mean annual precipitation is 340.2 mm, with the majority (86%) of the rainfall occurring during the growing season (May to September) during the previous 24 years (1982–2005). During the three experimental years (2006–2008), the mean annual temperature was 1.4°C, 2.2°C, and 1.6°C, respectively, and the annual total rainfall was 304.1 mm, 240.1 mm and 363.5 mm, respectively [33].

### Plant materials, experimental design and treatments

The experiment was conducted on *C. microphylla* (Fig. S1). There were four treatment groups: ambient CO_2_ without N addition (C), elevated CO_2_ without N addition (E), ambient CO_2_ with N addition (CN), and elevated CO_2_ with N addition (EN). The experiment followed a split-plot design, with the CO_2_ treatment applied at the whole plot level (with three chambers for each of the two CO_2_ levels) and the N addition treatments applied at the split-plot level (pot-within-chamber). The experiment was conducted for three years (2006–2008) and most variables were measured in each year.

Six field open-top chambers (3 m in diameter, 3 m in height) were used. Three of the chambers contained the current CO_2_ concentration (380 µmol mol^−1^) and the other three contained an elevated CO_2_ concentration (760 µmol mol^−1^) (Fig. S2) [33]. For N addition treatment, 17.5 g m^−2^ of N [34] was added in each year by applying (NH_4_)_2_SO_4_ solution at the beginning and in the middle of the growing season.

The experiment site (15 m×15 m) was established in 2005. Pots (30 cm diameter, 30 cm deep) were filled with the universal native dark chestnut soil, and then buried underground with the pot mouth positioned at the ground surface. The soil organic carbon concentration was 0.081% and total N concentration was 0.704%. Seeds of *C. microphylla*, collected in the vicinity of the research station, were sown in pots in late autumn in 2005. At the beginning of the experiment in 2006, plants were thinned to 20 plants per pot.

Shoot biomass was harvested and oven dried at 65°C to constant mass and weighed in 2006–2007. In 2008, the whole plant was harvested, and shoot and root biomass processed separately. Soil was carefully removed from roots, which were temporarily stored at 4°C until all nodules could be removed for determination of symbiotic nitrogenase activity (see below). The remaining root biomass and shoot biomass were oven dried at 65°C to constant mass and weighed.

Symbiotic nitrogenase activity was determined by the C_2_H_2_ reduction method [35] using nodules within 48 h of collection. Nodules were placed in a 25 ml closed culture bottle and sealed with rubber. Three ml C_2_H_2_ gas was injected into the culture bottles and incubated for 2 hours at 28°C, after which 1 ml gas samples were collected from each bottle and analyzed for production of C_2_H_4_ using gas chromatography (Shimadzu GC-7AG gas chromatograph, Shimadzu Corp., Japan). The parameters used for spectrometer determination of symbiotic nitrogenase activity with the flame ionization detector (FID) were as follows: column temperature 60°C, detector temperature 250°C, sample temperature 120°C, gas flow of H_2_ 0.7 kg cm^−2^, N_2_ 35 ml min^−1^, and air 0.6 kg cm^−2^. Production of C_2_H_4_ (µmol g^−1^ h^−1^) was used to calculate symbiotic nitrogenase activity. Specific symbiotic nitrogenase activity (SNA) represented the symbiotic nitrogenase activity per unit weight of nodule. Plant symbiotic nitrogenase activity (PNA) represented the symbiotic nitrogenase activity per plant.
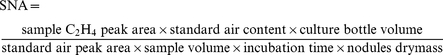


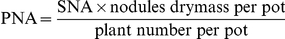



Specific symbiotic nitrogenase activity for the CN treatment was treated as missing data since live root nodules collected from this treatment were insufficient for measurement.

Leaf gas exchange (µmol CO_2_ m^−2^ s^−1^) measurements were conducted *in situ* using a portable, steady-state gas exchange system, incorporating an infrared gas analyzer (LI-6400, Li-Cor, Lincoln, NE, USA), in late August 2007 and mid-July 2008. Measurements were made on three leaves of randomly chosen individual plants in each pot on sunny days. The second healthy leaf from the top of the plants was selected. Photosynthetic rates were determined under light-saturating conditions (PAR 1500 µmol m^−2^ s^−1^), constant leaf air temperature (25°C), and at the CO_2_ concentration under which the plants were grown (i.e., 380 or 760 µmol mol^−1^) as long-term responses. In addition, for plants grown under ambient CO_2_ photosynthetic rates were determined at 760 µmol mol^−1^ CO_2_ to assess the short-term response of the species to instantaneous CO_2_ enrichment [36]. Following gas exchange measurement, leaf areas were measured with a leaf area meter (LI-3100, Li-Cor) and photosynthetic rates were calibrated with the known area.

### Statistical analysis

Statistical analysis of data was conducted using the Statistical Analysis System 9.2 (SAS Institute Inc., Cary, NC, USA). There were three replicates for each treatment group. Split-plot analysis of variance (ANOVA) was performed using mixed model procedures with CO_2_ concentration as the whole-plot factor and N as the split-plot factor. Year was taken as a repeated factor for indices measured in multiple years, such as photosynthetic rate, shoot biomass, and leaf N content. Tukey's studentized range test was conducted to make pairwise comparisons of means for those indices in which ANOVA showed a significant effect. Results were considered to be significant at *P*≤0.05, and highly significant at *P*≤0.01.

## Results

### Effects of elevated CO_2_ and N addition on net photosynthesis

**Figure 1:**
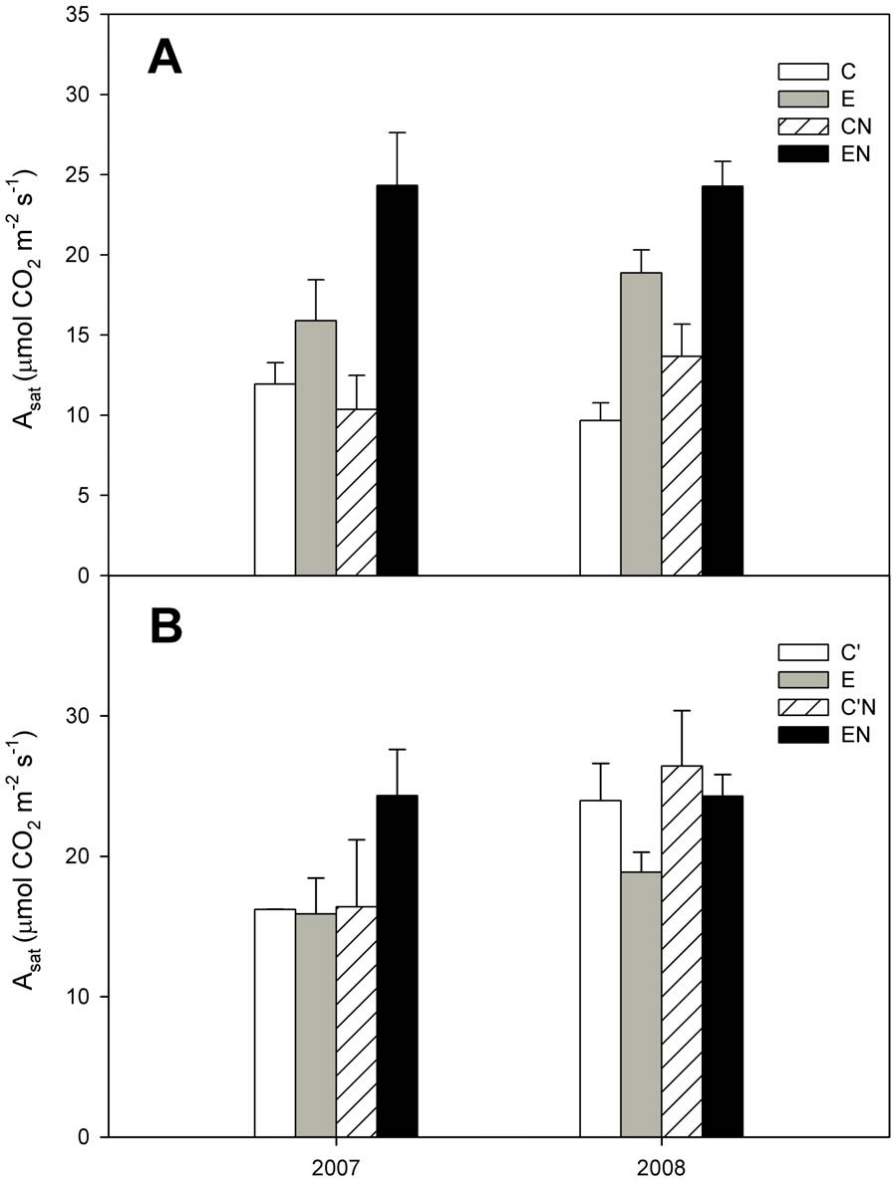
Light-saturated leaf net photosynthetic rate (A_sat_, µmol m^−2^ s^−1^) and down-regulation of photosynthesis of *C. microphylla*. Plants were grown and measured under ambient (380 µmol mol^−1^) or elevated (760 µmol mol^−1^) CO_2_ concentrations (A), and plants were grown under ambient or elevated CO_2_ and measured at a common CO_2_ concentration of 760 µmol mol^−1^ (B) at two nitrogen-amended levels (0 and 17.5 g N m^−2^ year^−1^ applied to native N-deficient soil) in the 2007 and 2008 growing seasons. Treatments: ambient CO_2_ without N addition (C); elevated CO_2_ without N addition (E); ambient CO_2_ with N addition (CN); elevated CO_2_ with N addition (EN); grown at ambient but measured in elevated CO_2_ without N addition (C′); grown at ambient but measured in elevated CO_2_ with N addition (C′N). Bars represent means and error bars the standard error.

Both elevated CO_2_ and N addition had significant effects on leaf-level gas exchange of *C. microphylla* (*P*<0.05, Table 1), and their interactions also had a significant effect (*P*<0.01, Table 1). Elevated CO_2_ markedly increased the light-saturated leaf net photosynthetic rate (A_sat_) under both N levels across the three years, with average increases of 83% (Fig. 1A). Plant grown under high N showed an average 29% higher photosynthetic rate than those grown under low N. However, the stimulatory effect of N addition on A_sat_ was different between the two CO_2_ concentrations, with a 40% increase occurring at the elevated CO_2_ concentration. The responses of A_sat_ to elevated CO_2_, N addition or both did not vary significantly between the 2 years (Table 1).

**Table 1:** Results (*P*-values) of mixed model ANOVA for the effects of elevated CO_2_ (CO_2_), N addition (N) and their interactions on shoot biomass and leaf nitrogen content (Leaf N) in three growing years (Y; 2006 to 2008), and light-saturated leaf net photosynthetic rate (A_sat_) and down-regulation of photosynthesis (D) in 2007 and 2008, and root biomass, root/shoot ratio (R/S), specific symbiotic nitrogenase activity (SNA) and symbiotic nitrogenase activity per plant (PNA) in 2008.

Source of variation	A_sat_	D	Shoot biomass	Leaf N	Root biomass	R/S	SNA	PNA
CO_2_	0.01	0.94	0.03	0.52	0.19	0.69	0.86	0.74
N	<0.01	0.01	0.30	<0.01	0.11	<0.01	0.89	0.01
CO_2_×N	0.01	0.05	0.04	0.53	0.39	<0.01	/	0.64
Y	0.55	0.07	<.0001	<.001				
Y×CO_2_	0.78	0.17	0.16	0.41				
Y×N	0.70	0.94	0.57	0.02				
Y×CO_2_×N	0.21	0.61	0.20	0.34				

In addition, when measured with the same elevated CO_2_, A_sat_ did not differ significantly between C plants and E plants, as well as between CN plants and EN plants. This implied that no acclimation of photosynthesis occurred in *C. microphylla* (Table 1, Fig. 1B).

### Effects of elevated CO_2_ and N addition on aboveground growth

**Figure 2:**
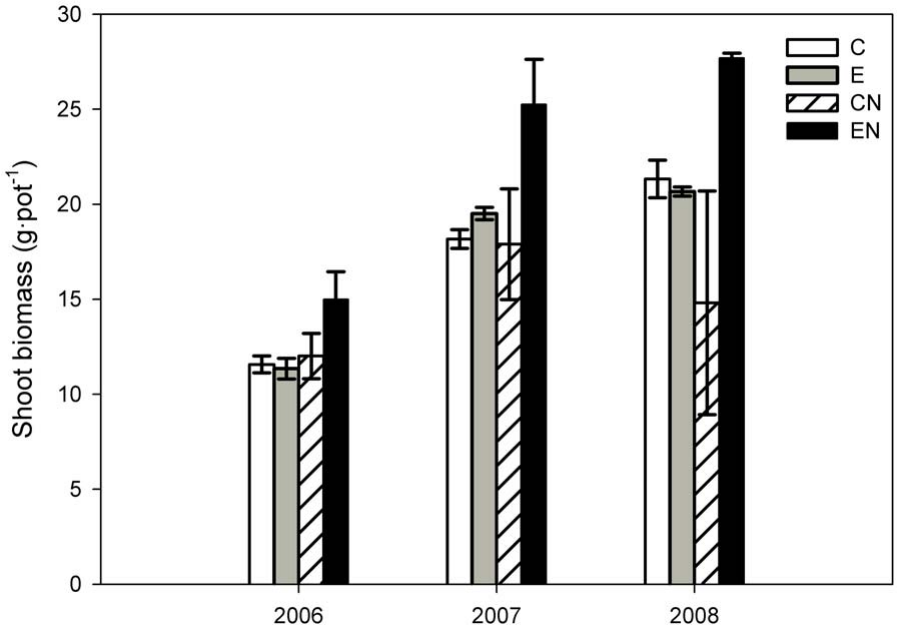
Shoot biomass per pot of *C. microphylla*. Plants were grown in open-top chambers under ambient (380 µmol mol^−1^) and elevated (760 µmol mol^−1^) atmospheric CO_2_ concentrations at two nitrogen levels (0 and 17.5 g N m^−2^ year^−1^) in the growing seasons from 2006 to 2008. Treatments: ambient CO_2_ without N addition (C); elevated CO_2_ without N addition (E); ambient CO_2_ with N addition (CN); elevated CO_2_ with N addition (EN). Bars represent means and error bars the standard error.

Elevated CO_2_ significantly stimulated shoot biomass by 25% when all treatments were considered (*P*<0.05, Table 1). The stimulation of elevated CO_2_ was significant only under high N concentration (Fig. 2). Addition of N significantly stimulated shoot biomass by 32% (*P*<0.05) at elevated CO_2_, but showed no significant effect on shoot biomass at ambient CO_2_ across the three years.

Shoot biomass in 2006 was significantly lower than in 2007 and 2008. The shoot biomass increased by 62% (*P*<0.001) in 2007 and 69% (*P*<0.001) in 2008 compared with that in 2006. Annual variation in shoot biomass was not significantly affected by CO_2_ concentration, N addition or their interaction.

### Effects of elevated CO_2_ and N addition on root biomass and root/shoot ratio

**Figure 3:**
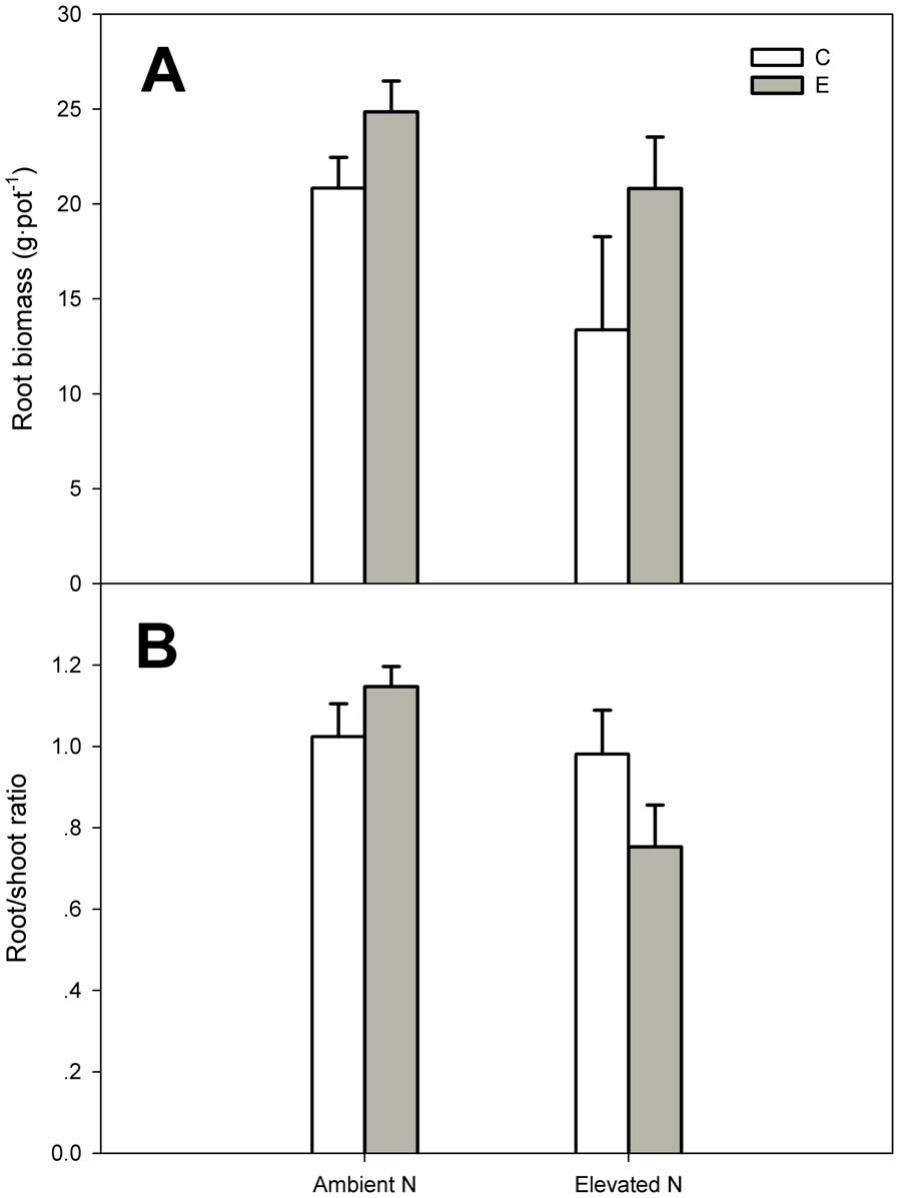
Root biomass (A) and root/shoot ratio (B) per pot of *C. microphylla*. Plants were grown under ambient (380 µmol mol^−1^) and elevated (760 µmol mol^−1^) atmospheric CO_2_ concentrations at two nitrogen levels (0 and 17.5 g N m^−2^ year^−1^) in 2008. Treatments: ambient CO_2_ without N addition (C); elevated CO_2_ without N addition (E); ambient CO_2_ with N addition (CN); elevated CO_2_ with N addition (EN). Bars represent means and error bars the standard error.

Neither elevated CO_2_, N addition nor their interaction significantly affected root biomass (Fig. 3A, Table 1). Elevated CO_2_ had no significant effect on root/shoot ratio either. However, N addition significantly decreased the root/shoot ratio by 34% at elevated CO_2_ (*P*<0.01, Table 1), but had no significant effect on the root/shoot ratio at ambient CO_2_ (Table 1, Fig. 3B).

### Effects of elevated CO_2_ and N addition on specific symbiotic nitrogenase activity and symbiotic nitrogenase activity per plant

**Figure 4:**
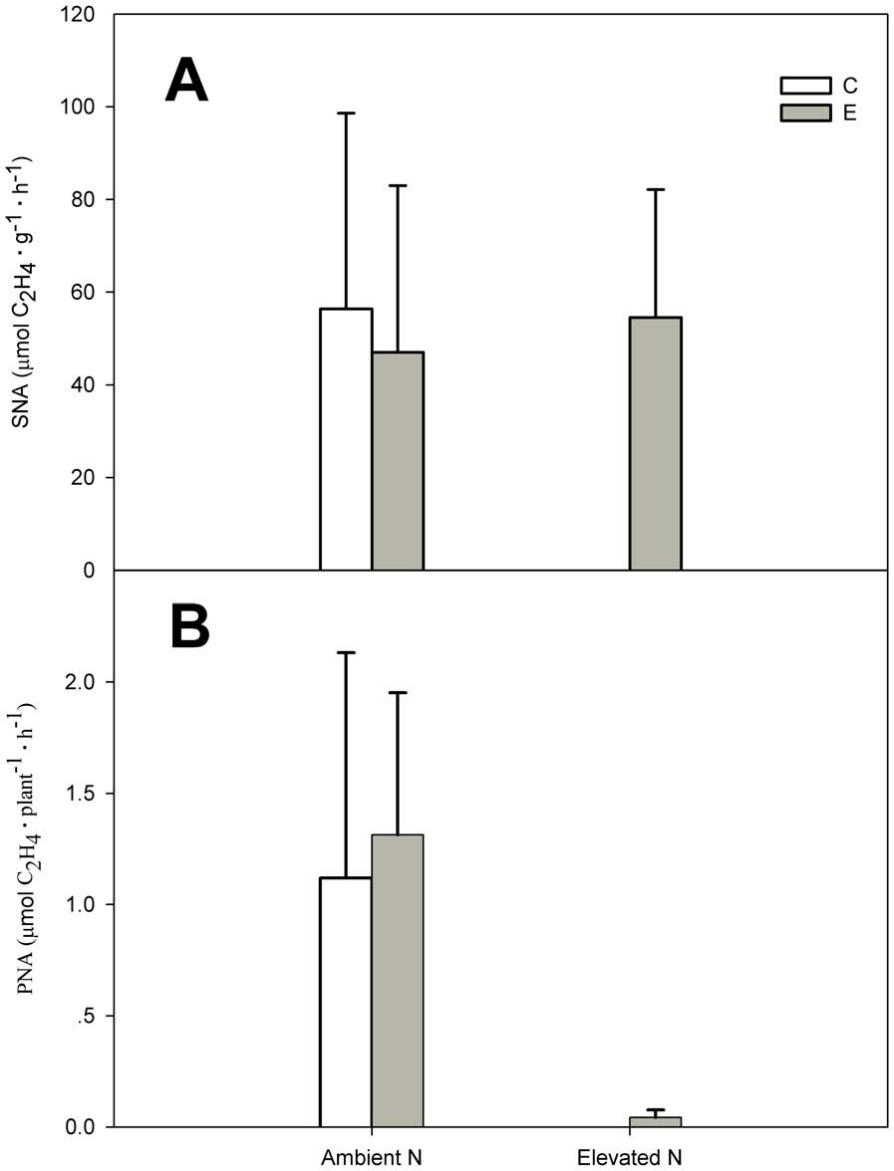
The specific symbiotic nitrogenase activity (SNA, µmol C_2_H_2_ g^−1^ h^−1^) (A), and symbiotic nitrogenase activity per plant (PNA, µmol C_2_H_2_ g^−1^ h^−1^) (B) of *C. microphylla*. Plants were grown under ambient (380 µmol mol^−1^) and elevated (760 µmol mol^−1^) atmospheric CO_2_ concentrations at two nitrogen levels (0 and 17.5 g N m^−2^ year^−1^) in 2008. Treatments: ambient CO_2_ without N addition (C); elevated CO_2_ without N addition (E); ambient CO_2_ with N addition (CN); elevated CO_2_ with N addition (EN). Bars represent means and error bars the standard error.

Elevated CO_2_ did not significantly affect specific symbiotic nitrogenase activity (Fig. 4A) and symbiotic nitrogenase activity per plant (Fig. 4B, Table 1). Addition of N had no significant effect on specific symbiotic nitrogenase activity, but markedly decreased symbiotic nitrogenase activity per plant by over 95% (*P*<0.05, Table 1, Fig. 4).

### Effects of elevated CO_2_ and N addition on leaf N content

**Figure 5:**
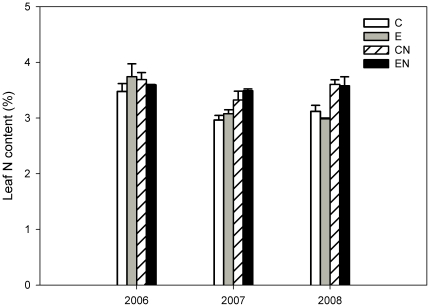
The leaf N content (Leaf N, %) of *C. microphylla*. Plants were grown under ambient (380 µmol mol^−1^) and elevated (760 µmol mol^−1^) atmospheric CO_2_ concentrations at two nitrogen levels (0 and 17.5 g N m^−2^ year^−1^) in the growing seasons from 2006 to 2008. Treatments: ambient CO_2_ without N addition (C); elevated CO_2_ without N addition (E); ambient CO_2_ with N addition (CN); elevated CO_2_ with N addition (EN). Bars represent means and error bars the standard error.

Elevated CO_2_ had no significant effect on leaf N content, whereas N addition significantly enhanced leaf N content by 10% in all treatments over the three years (*P*<0.01, Table 1, Fig. 5). No interaction effect between CO_2_ and N addition on leaf N content was detected. The increase in leaf N content induced by N addition differed between the three years: 1% in 2006 (ns), 13% in 2007 (*P*<0.01) and 18% in 2008 (*P*<0.01).

## Discussion

### Effect of elevated CO_2_ on N_2_ fixation and growth of *C. microphylla*

Our finding that elevated CO_2_ has a stimulatory effect on photosynthetic rates in the leguminous subshrub *C. microphylla* is consistent with most previous studies on other plant species [37], [38], [39]. Numerous studies have found that stimulation of photosynthetic rates induced by elevated CO_2_ will decrease or even disminished over time as plants acclimate to elevated CO_2_ concentrations through a process known as down-regulation [36], [38], [40]. Acclimation of photosynthesis can be partly explained by N scarcity or progressive reduction in N availability, since the down-regulation of photosynthesis induced by elevated CO_2_ is always highly associated with reduction of leaf N content [5], [39]. Results in the present study revealed that acclimation of photosynthesis did not occur in *C. microphylla*. This is in line with the hypothesis proposed by many previous studies that species capable of symbiosis with N_2_-fixing organisms may sustain longer stimulation [2], [9]. Accordingly, leaf N content of *C. microphylla* in this study did not vary with CO_2_ concentration.

In the present study, symbiotic nitrogenase activity did not respond significantly to elevated CO_2_ concentrations. The effect of elevated CO_2_ on N_2_-fixation may be positive, neutral or negative, depending on the species examined, soil N acquisition [18], or availability of other soil resources, such as phosphorus [14], [22]. Thus, it can be inferred that the symbiotic N_2_-fixation of *C. microphylla* is not sensitive to changes in CO_2_ concentration. Another possible explanation is that other limited nutrients, such as P, constrain the responses of N_2_-fixation of *C. microphylla* to elevated CO_2_. Availability of P is reported to be more limited than that of N in our experimental area [41]. Although the N_2_-fixation was not enhanced by elevated CO_2_, *C. microphylla* could utilize soil N more efficiently at elevated CO_2_ (unpublished data).

The finding that elevated CO_2_ had a significant positive effect on shoot biomass, but not on root biomass, implies that shoot growth of *C. microphylla* is more sensitive to elevated CO_2_ than that of root growth. This finding is in contrast to the non-leguminous species *Leymus chinensis* in the same experiment field [33]. Previous studies have shown that the relative responses of root systems to elevated CO_2_ are species specific and dependent on experimental conditions [33], [42], [43]. For example, Arnone *et al.* found that among 12 grassland studies in different areas, seven showed little or no change in root-system size under elevated CO_2_ [44]. On the other hand, pots may constrain growth of the root system and may therefore partly depress the root response to elevated CO_2_ when plants are grown in pots [33], [45]. However, root growth of *C. microphylla* was not significantly suppressed by pot containment in this study because *C. microphylla* is a slow-growing shrub and was in early seedling stage. Additionally, the shoot response of *C. microphylla* to elevated CO_2_ is much higher than *L. chinensis* (25% vs. 9%) in the same experimental field [33]. This is in line with most previous studies, which reported that legume species show a stronger response to elevated CO_2_ than non-leguminous species [39]. This result implies that the relative competitiveness of the legume *C. microphylla* with *L. chinensis*, the dominant temperate grassland species in the study area, will increase in the future under elevated CO_2_ conditions.

In the present study, the stimulatory effect of elevated CO_2_ on *C. microphylla* was dependent on N status. This finding is similar to that with other shrubs [46], but in contrast to herbaceous legume species, which always show a strong response to elevated CO_2_ independent of N status [9]. On the other hand, the effect of CO_2_ on photosynthesis, growth, and leaf N content of *C. microphylla* did not show any annual variation, even though the weather conditions varied among the study years.

### Effect of N addition and its interaction with elevated CO_2_ on growth and N_2_ fixation of *C. microphylla*

The stimulatory effects of N addition on photosynthesis and shoot biomass, as well as its inhibitory effect on the root/shoot ratio, were only observed under elevated CO_2_ in the present study. These results indicate that accumulation of photosynthate and biomass allocation in response to soil N supply was affected by elevated CO_2_. The finding that N addition had no significant effect on biomass production of *C. microphylla* at ambient CO_2_ is consistent with some previous studies on legumes [47], but is inconsistent with the effect on biomass production by the grass species *L. chinensis* in the same experimental field, which was greatly increased by N addition. This indicated the competitiveness of *C. microphylla* with the herbaceous species *L. chinensis* would decrease with N addition at ambient CO_2_. However, when plants were grown under elevated CO_2_, N addition had a positive effect on *C. microphylla* growth and with a similar degree of enhancement on *L. chinensis*. This indicates that the depressive effect of N fertilization or deposition on the competitiveness of *C. microphylla* with *L. chinensis* would be alleviated by elevated CO_2_. This is in line with indications from other studies that elevated CO_2_ may reduce the increased risk of legume species loss due to the N fertilization or deposition [3], [48].

The most obvious effect of N addition on *C. microphylla* in the present study is its inhibitory effect on symbiotic nitrogenase activity per plant. This inhibitory effect is in agreement with most previous reports on other plant species, while the detrimental effect of N addition on symbiotic nitrogenase activity per plant in the current study is more serious than that of other reports [9], [19]. The greater effect may be attributed to the relatively high N concentration applied in the present study. Given that no changes in specific symbiotic nitrogenase activity and reduction in root nodule number under the high N level in the current study [49], it can be concluded that the strong decrease in symbiotic nitrogenase activity per plant under the high N level mainly resulted from the inhibitory effect of N addition on nodule formation.

In the present study, the effect of N addition on photosynthesis and shoot biomass of *C. microphylla* did not show interannual variation. However, the stimulatory effect of N addition on leaf N content was increased across years. This implies that the responses of *C. microphylla* to N addition may be more affected by interannual climatic variation than elevated CO_2_.

In conclusion, we demonstrated that elevated CO_2_ stimulates leaf-level photosynthesis of *C. microphylla* and no acclimation of photosynthesis occurred over the three experimental years. Elevated CO_2_ stimulates shoot growth of *C. microphylla*, but only under a high N concentration. Shoot growth of *C. microphylla* is more sensitive to elevated CO_2_ than is root growth. Elevated CO_2_ has no effect on symbiotic nitrogenase activity. Addition of N markedly inhibits N_2_ fixation capacity, but stimulates photosynthesis and shoot growth, of *C. microphylla*. However, the stimulatory effect of N addition occurred only under elevated CO_2_ condition. Interaction between CO_2_ and N significantly affected photosynthesis, shoot biomass and biomass allocation of *C. microphylla*. When compared to responses of the grass species *L. chinensis* grown in the same experimental field, N addition tends to decrease the relative competitiveness of *C. microphylla*, whereas elevated CO_2_ tends to increase competitiveness. These results indicate that elevated CO_2_ will interact with N deposition in the future to benefit the growth of the leguminous subshrub *C. microphylla*.

## Supporting Information

Figure S1 The photograph of the *C. microphylla* in the Xilin River Basin.
(TIF)

Figure S2 The photograph of the six open-top chambers.
(TIF)
