# Supplementary figures and images for: Effects of Elevated CO2 and N Addition on Growth and N2 Fixation of a Legume Subshrub (Caragana microphylla Lam.) in Temperate Grassland in China
Source: PLoS One. 2011 Oct 26;6(10):e26842. doi: 10.1371/journal.pone.0026842 (PMC3202592; doi:10.1371/journal.pone.0026842)

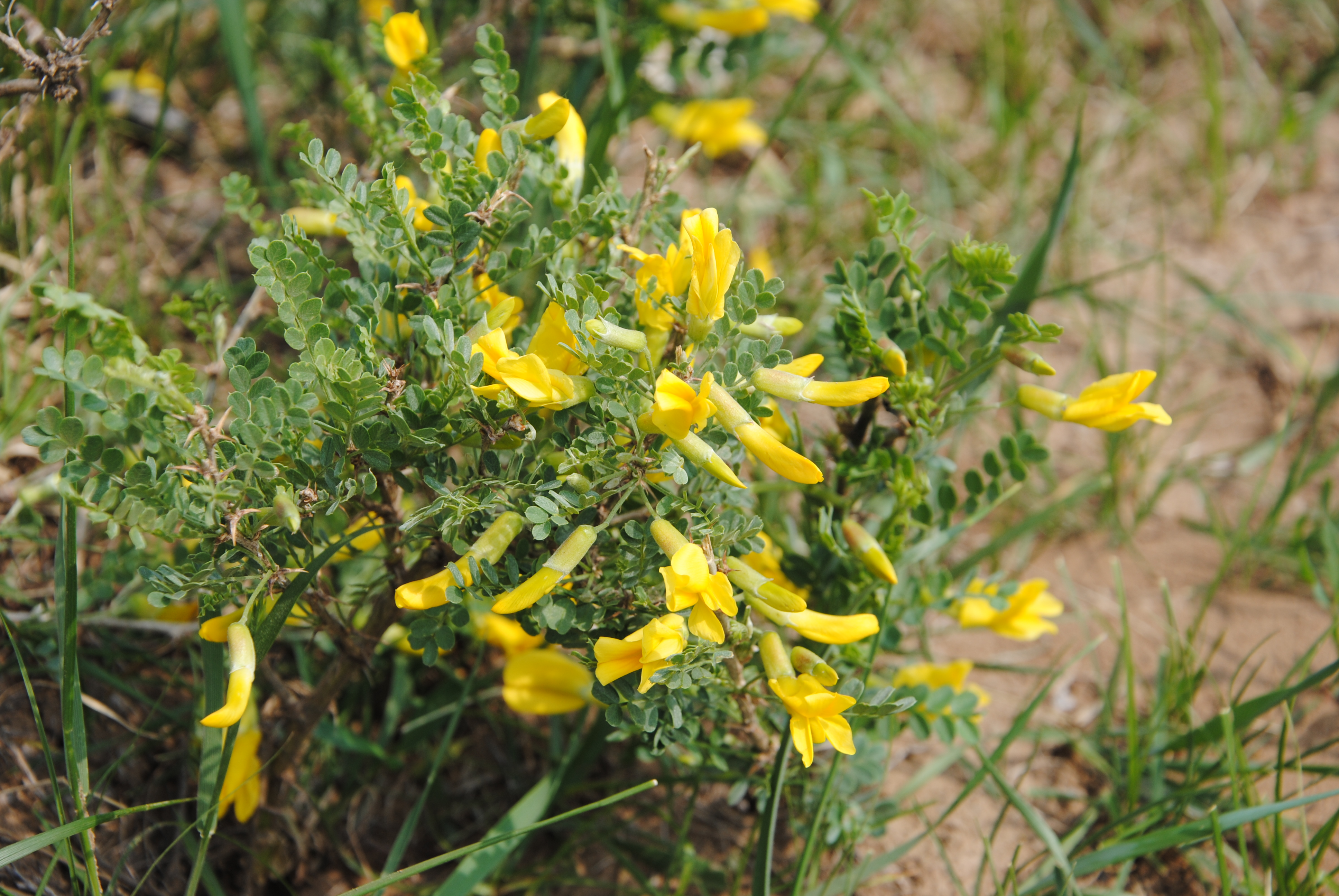

Supplement: Figure S1 — The photograph of the C. microphylla in the Xilin River Basin. (TIF) [file pone.0026842.s001.tif]

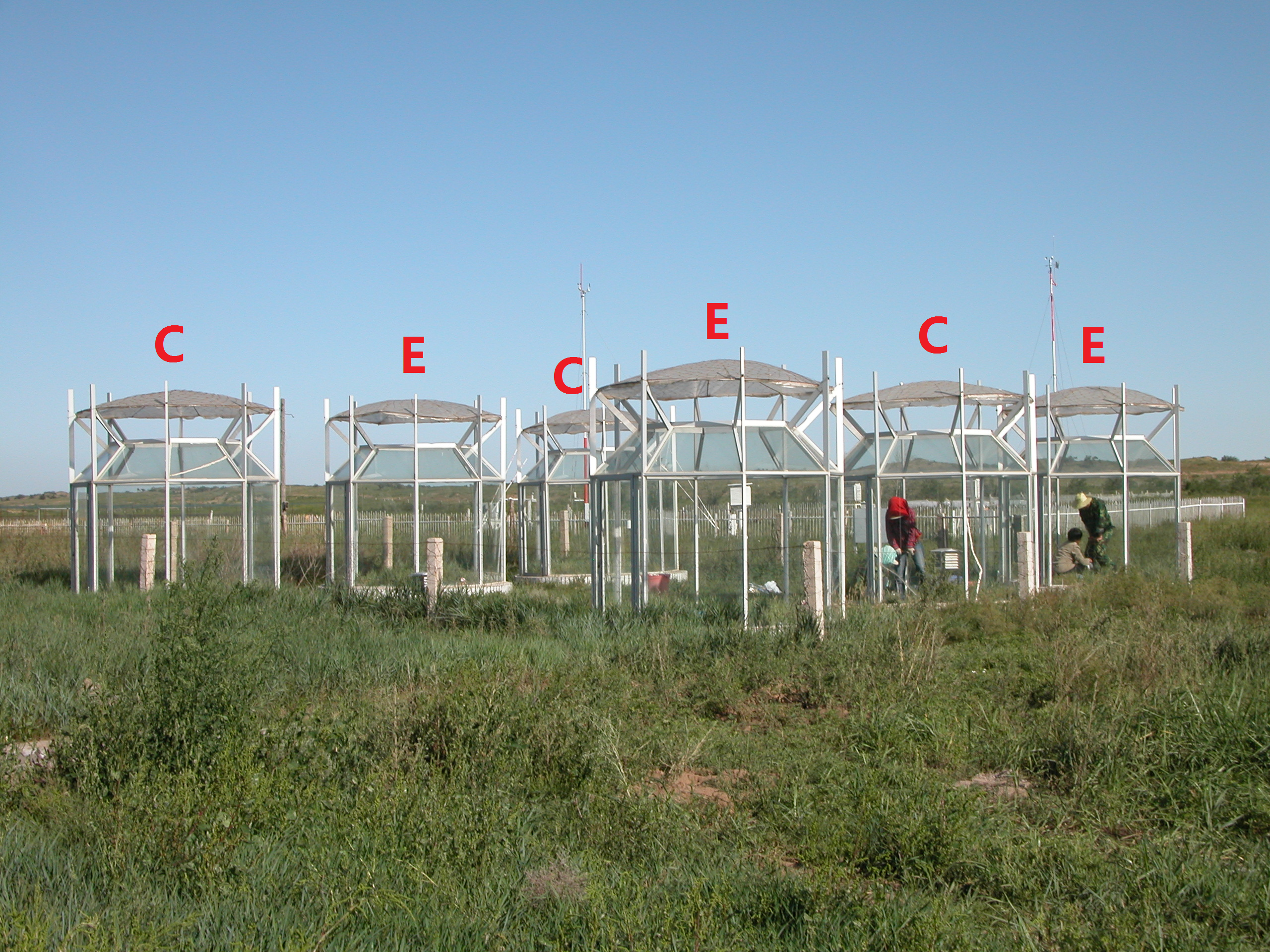

Supplement: Figure S2 — The photograph of the six open-top chambers. (TIF) [file pone.0026842.s002.tif]
